# Adding loci improves phylogeographic resolution in red mangroves despite increased missing data: comparing microsatellites and RAD-Seq and investigating loci filtering

**DOI:** 10.1038/s41598-017-16810-7

**Published:** 2017-12-14

**Authors:** Richard G. J. Hodel, Shichao Chen, Adam C. Payton, Stuart F. McDaniel, Pamela Soltis, Douglas E. Soltis

**Affiliations:** 1 Department of Biology University of Florida, Gainesville, FL 32611 USA; 2 Florida Museum of Natural History University of Florida, Gainesville, FL 32611 USA; 3 College of Life Sciences and Technology Tongji University, Shanghai, 200092 China; 4 The Genetics Institute University of Florida, Gainesville, FL 32610 USA

## Abstract

The widespread adoption of RAD-Seq data in phylogeography means genealogical relationships previously evaluated using relatively few genetic markers can now be addressed with thousands of loci. One challenge, however, is that RAD-Seq generates complete genotypes for only a small subset of loci or individuals. Simulations indicate that loci with missing data can produce biased estimates of key population genetic parameters, although the influence of such biases in empirical studies is not well understood. Here we compare microsatellite data (8 loci) and RAD-Seq data (six datasets ranging from 239 to 25,198 loci) from red mangroves (*Rhizophora mangle*) in Florida to evaluate how different levels of data filtering influence phylogeographic inferences. For all datasets, we calculated population genetic statistics and evaluated population structure, and for RAD-Seq datasets, we additionally examined population structure using coalescence. We found higher *F*_*ST*_ using microsatellites, but that RAD-Seq-based estimates approached those based on microsatellites as more loci with more missing data were included. Analyses of RAD-Seq datasets resolved the classic Gulf-Atlantic coastal phylogeographic break, which was not significant in the microsatellite analyses. Applying multiple levels of filtering to RAD-Seq datasets can provide a more complete picture of potential biases in the data and elucidate subtle phylogeographic patterns.

## Introduction

Choice of molecular markers remains a critically important consideration when designing a phylogeographic, phylogenetic, or population genetic study, as researchers must optimize the amount of informative genetic data they can obtain for a fixed and typically modest cost. In phylogeographic studies, theoretical considerations impact decisions regarding whether to include more individuals or more loci. Microsatellites (or simple sequence repeats, SSRs) have been one of the workhorses of phylogeographic studies for over two decades—their high variability made them popular for distinguishing between closely related conspecific or congeneric individuals^1–3^. Microsatellite markers are now being gradually replaced by RAD-Seq data for phylogeographic inference^4^.

There are advantages and disadvantages to using microsatellites in phylogeographic studies^2,3,5^. Microsatellites are a known quantity; hundreds of thousands of studies that use SSRs are in the literature—primers are already available for many groups. In addition, many user-friendly software packages are available for all aspects of microsatellite analysis, from loci development to population genetic inference^3^. If primers are already developed for the taxa of interest, microsatellites can be inexpensive to implement. Additionally, if initial results necessitate adding a few additional individuals and/or loci, project costs will increase linearly with microsatellites. However, there are caveats to using SSRs. Perhaps most importantly, a limited number of loci (usually < 25) can feasibly be employed in a typical microsatellite study. Also, the mutational properties of SSRs are unusually high and almost certainly do not reflect those of the genome as a whole. Thus, the property that makes microsatellites excellent for distinguishing different individuals may inflate statistics such as *F*_*ST*_ and heterozygosity relative to the rest of the genome. Furthermore, microsatellites can be just as expensive to implement as newer high-throughput sequencing (HTS) techniques if there are no existing genetic resources (e.g., no primers already developed, or no available transcriptomic or genomic resources)^6^.

The use of RAD-Seq data has increased greatly over the past decade, largely because thousands of loci can be generated simultaneously for hundreds of individuals for a fixed, known cost^7^. RAD-Seq uses restriction enzymes (REs) to create a reduced representation library of the genome; single-nucleotide polymorphisms (SNPs) in regions of DNA between restriction sites are used to distinguish between individuals^8^. Barcoding to allow efficient multiplexing during sequencing keeps costs down, which can be as little as $40 per individual for thousands of loci, assuming judicious sharing of reagents, and a well-designed plan for multiplexing individuals^9–11^. Microsatellite genotyping has a similar cost per individual, assuming primers are not developed, but many fewer loci are obtained^3^. SNPs have several advantages over microsatellites, as they are less likely to exhibit homoplasy than SSRs^12^.

Despite advantages, there are also several caveats to using RAD-Seq. Unless there is a reference genome, loci obtained using RAD-Seq are anonymous, and some loci may be non-neutral^7^. Additionally, biases may be introduced at several stages in a RAD-Seq protocol: (1) digestion with REs samples a non-random portion of the genome due to biases in base composition; this is potentially worse if methylation sensitive enzymes are used; (2) polymorphisms in restriction sites that can lead to segregating presence/absence polymorphisms that are very difficult to detect without very deep sequencing and negating the cost-savings of using RAD-Seq in the first place^7,13^; (3) preferential PCR amplification of some loci during library construction necessarily reduces coverage of other loci^13^; (4) sequencing errors and/or low sequencing depth leads to incorrect genotype calling^7^; and (5) false loci are constructed due to the misassembly of paralogous reads^14,15^. Many potential problems are resolved by multiple PCR steps to even out loci coverage and by improvements in software when processing loci, but concerns remain that RE-based reduced representation methods do not capture a representative snapshot of the genome^16^. One other concern with RAD-Seq loci is that manual data curation is impossible, and errors may go undetected even by the most careful researchers^14,17,18^. Finally, the biggest potential problem when using RAD-Seq is that low coverage and high proportions of missing data can make it difficult to infer heterozygotes accurately.

Previous studies have compared results from SNPs and SSRs, revealing that microsatellites provide much more information—up to an order of magnitude more—on a per-marker basis than SNPs^19,20^. However, SNP studies typically use several orders of magnitude more markers than an average SSR study. Evidence has shown that the large number of loci in SNP studies can effectively allow for more powerful inferences, even though the information at each locus is less than that in microsatellite markers^21^. Because of the low number of loci used in SSR studies, the standard practice is to aim to minimize missing data. However, the nature of current library preparation and sequencing means that higher percentages of missing data are an unavoidable part of RAD-Seq studies. Simulation studies have shown that the large amounts of missing data in RAD-Seq studies can inflate *F*_*ST*_ estimates due to allelic dropout^13,18^. As more loci were included in these simulations, *F*_*ST*_ appeared to increase because many loci had genotype data for only one or a few individuals. In many such loci *F*_*ST*_ = 1 because by chance the few individuals sampled were homogeneous within populations but different between populations, leading to high average *F*_*ST*_. Heterozygosity can be similarly inflated if the more frequent allele is likely to be absent (e.g., because mutations in the restriction site, which lead to allelic dropout, are often in ancestral alleles that occur at a high frequency)^18^. Arnold, *et al*.^13^ confirmed results from Gautier, *et al*.^18^ and also concluded that other summary statistics, including Θ and π, could be inaccurately estimated from loci with missing data. In spite of these problems, more recent simulation studies have indicated that missing data in RAD-Seq studies may not lead to incorrect inference, and in fact including loci with missing data can be advantageous for identifying shallow divergences^22^.

Convention in phylogeographic studies often is to require 75 or 80% of individuals to have data for a given locus—otherwise that locus is discarded from the analyses (e.g., refs^23–28^). Presumably, requiring a locus to be present in a certain number of individuals will eliminate loci with high missing data that may be the cause of misestimated parameters^13,18^. However, the choice of a cutoff is arbitrary and is typically not justified in phylogeographic studies— the number of SNPs is virtually always reported as a single fixed value (e.g., “we identified a total of 4,234 SNPs,” Jackson, *et al*.^24^). In reality, the various parameter values that determine how many loci are constructed and retained in SNP alignment methods means that there is a range of loci that could conceivably be included in a study^27,29^.

To date, no phylogeographic study has investigated the effect of varying amounts of missing data in an empirical RAD-Seq dataset, even those explicitly comparing RAD-Seq-generated SNPs and microsatellites^30,31^. To remedy this knowledge gap, we investigate the phylogeography of red mangroves (*Rhizophora mangle* L., Rhizophoraceae) in Florida, using both an existing microsatellite dataset^32^, and new RAD-Seq SNP datasets that vary in number of loci and the percentage of missing data. We filtered RAD-Seq loci to generate a dataset that would approximate the number of loci and amount of missing data typically used in RAD-Seq phylogeography studies, and we also generated datasets with more or less stringent filtering to test the effects of increasing or decreasing the number of loci and percentage of missing data. Specifically, we address the following questions: (1) In RAD-Seq datasets, how are phylogeographic inferences affected by the number of loci used? (2) In RAD-Seq datasets, how are phylogeographic inferences affected by the percentage of missing data? (3) What are the important differences in performance between microsatellites and RAD-Seq data in population genetic and phylogeographic inference? (4) Do RAD-Seq data reveal any novel phylogeographic inferences not already recovered by microsatellites for red mangroves in Florida?

**Figure 1:**
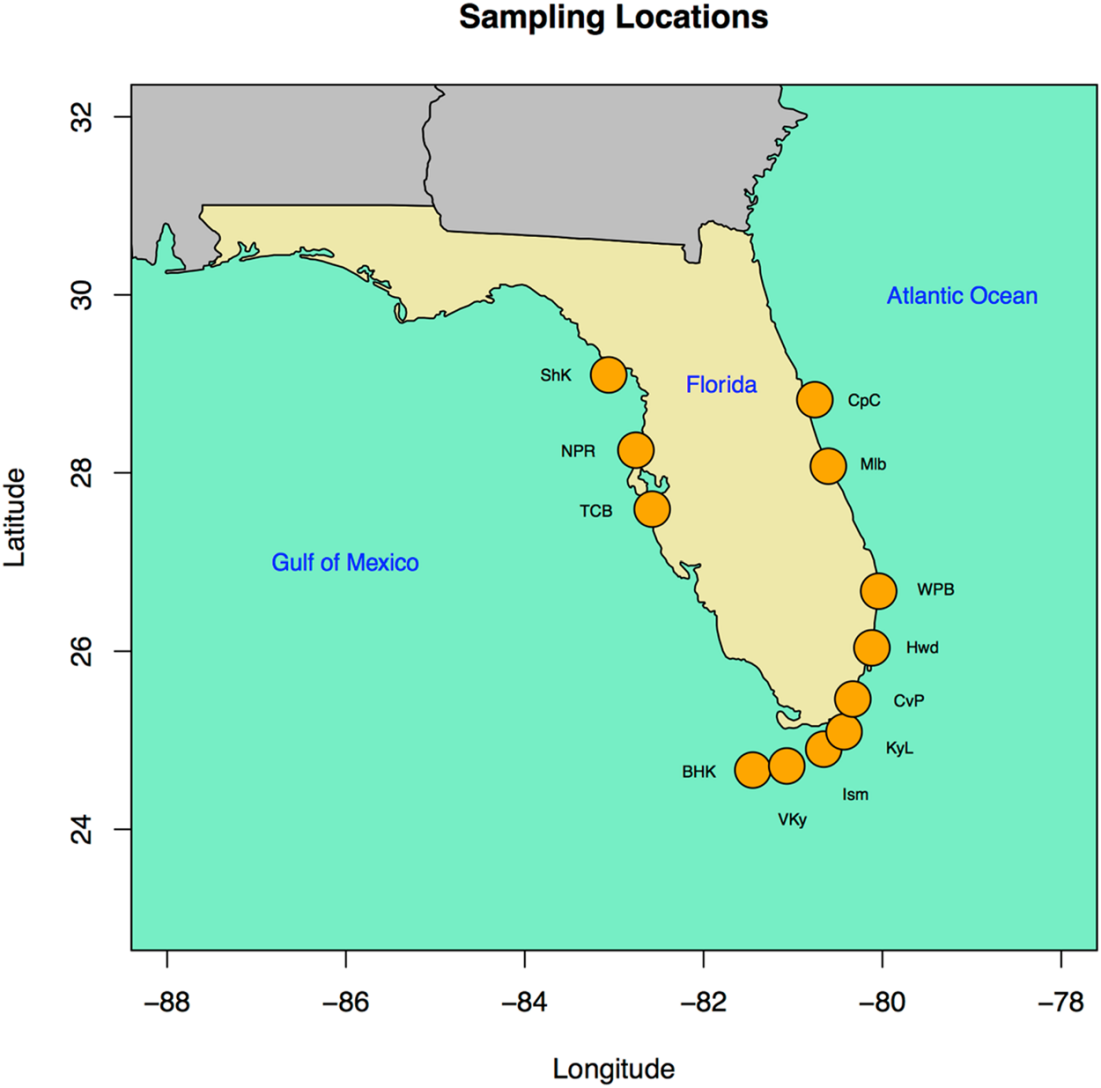
The 12 sampling locations (each with eight individuals) are indicated by orange circles. Sampling location codes are provided in Table 1. The map was generated using R (citation: R Core Team (2013). R: A language and environment for statistical computing. R Foundation for Statistical Computing, Vienna, Austria. http://www.R-project.org/), and the R package ‘maps’ (citation: Original S code by Richard A. Becker, Allan R. Wilks. R version by Ray Brownrigg. Enhancements by Thomas P Minka and Alex Deckmyn. (2017). maps: Draw Geographical Maps. R package version 3.2.0. https://CRAN.R-project.org/package=maps).

To address these questions, we used 96 red mangrove (*Rhizophora mangle*) individuals collected from 12 sampling locations on the coasts of Florida (Table 1, Fig. 1). Red mangroves are salt-tolerant trees that occur in coastal estuarine environments throughout the neotropics, experiencing high temperatures, frequent inundation, saline conditions, and periodic wave action associated with the coastal environment^33^. Red mangroves provide a variety of ecosystem services, including filtering water, providing habitat to animals, stabilizing shorelines, and protecting coastal environments from frequent wave action and occasional storm surges. Thus, red mangroves are important conservation targets—for which phylogeographic data can improve conservation strategies—making red mangroves a valuable study system.

**Table 1:** The twelve sampling locations (each containing eight individuals), their codes, GPS coordinates, and the percentage of loci that have missing data for each sampling location before any filtering.

Sampling Location	Code	Latitude (N)	Longitude (W)	% Loci Missing
Bahia Honda Key	BHKFl	24.55286	81.76776	73.5
Convoy Point	CvPFl	25.46347	80.33133	81.2
Cape Canaveral	CpCFl	28.82173	80.75594	83.0
Hollywood	HwdFl	26.03841	80.11780	79.4
Islamorada	IsmFl	24.90031	80.65690	81.0
Key Largo	KyLFl	25.09569	80.42957	88.9
Melbourne	MlbFl	28.07435	80.60526	79.8
New Port Richey	NPRFl	28.25432	82.75723	69.5
Seahorse Key	ShKFl	29.10040	83.06185	65.8
Terra Ceia Bay	TCBFl	27.59172	82.57524	81.7
Vaca Key	VKyFl	24.71154	81.06992	85.1
West Palm Beach	WPBFl	26.67505	80.04259	83.9

Further analysis of phylogeographic patterns in red mangroves and other species occurring in the Florida peninsula is also warranted. Although previous studies of many coastal and marine taxa revealed a phylogeographic discontinuity at or near the southern tip of Florida^34,35^, recent work on red mangroves using microsatellites failed to identify such a pattern^32,36^. Different types of molecular markers could reveal new phylogeographic insights, due to broader sampling of the genome, and provide a predictive framework for understanding how genetic variation in this iconic species will respond to climate change. Finally, red mangroves are an ideal system for comparing the performance of alternative genetic markers, given previous analyses of microsatellite loci^32,36,37^ and the size of the genome (approximately 1 Gb; Hodel unpublished data, based on flow cytometry observations), enabling a rigorous test of the RAD-Seq method. Genome size is a necessary consideration with RAD-Seq; as genome size increases, the number of loci shared among many individuals for a given sequencing depth decreases.

## Results

### Datasets

Seven datasets, ranging from 8 loci (SSR_8) to 25,198 loci (RAD_25198; Table 2), were used to investigate in depth how variation in number of loci and percent missing data impacted phylogeographic inferences. These were selected from all possible datasets, for which basic statistics were calculated (Supplementary Figure 1). The name of each of the seven datasets contains information about locus type (RAD or SSR) and number of loci in the dataset. The smallest RAD dataset contained 239 loci (RAD_239), and the percentage of missing data for RAD datasets ranged from 11.7% to 78.1% (Table 3). The dataset RAD_1180, which required a locus to be present in 75 of 96 individuals (78.1%), most closely mimicked the amount of loci filtering typically used in a phylogeographic study. Therefore, in our analyses, we used this as a baseline dataset against which to compare other RAD datasets. Across sampling locations, the proportion of missing data was relatively uniform (Table 1); percentage of missing loci in the data matrix for a given sampling location ranged from 65.8% to 88.9%.

**Table 2:** The seven data sets used in this study; RAD-Seq data sets were generated by filtering loci from largest data set (RAD_25198). For all data sets (six RAD and one microsatellite), the total number of loci used is indicated.

Dataset	Individuals required to retain a locus	Number of loci	% individuals required to retain a locus
RAD_239	83	239	86.5
RAD_1180	75	1180	78.1
RAD_2317	65	2317	67.7
RAD_3831	50	3831	52.1
RAD_6255	30	6255	31.3
RAD_25198	1	25198	1.0

**Table 3:**
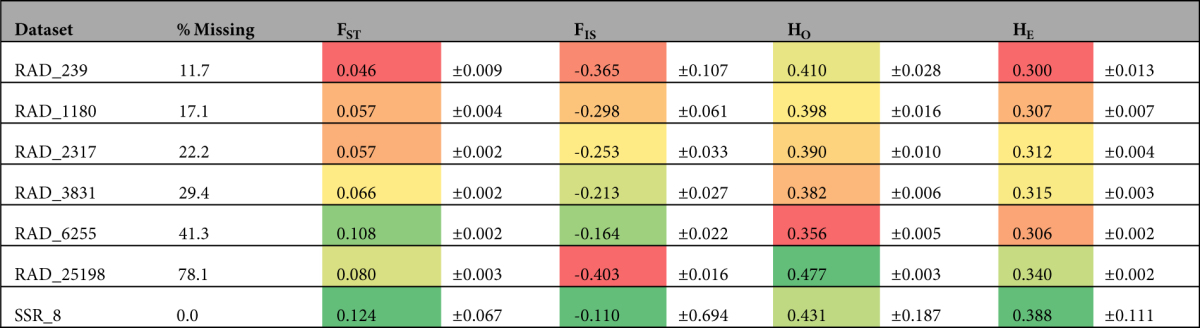
Relevant population genetic statistics for each of the seven data sets used in this study. For each column, warmer colors indicate lower values and cooler colors show higher values. Immediately to the right of each of the four columns (*F*_*ST*_, *F*_*IS*_, *H*_*O*_, *H*_*E*_) is the 95% confidence interval for each statistic.

### Population genetic analyses

**Figure 2:**
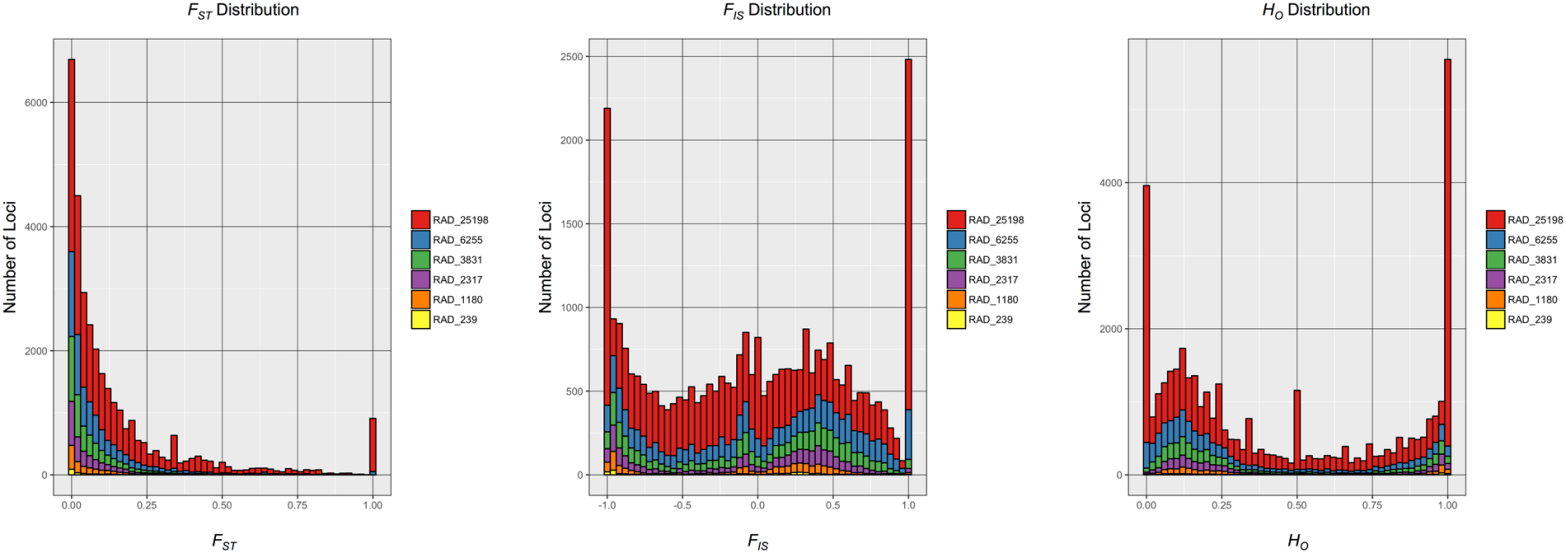
Stacked histograms of per locus estimates of *F*_*ST*_, *F*_*IS*_, and *H*_*O*_ for each of the RAD datasets. Datasets with more loci are stacked on top of datasets with fewer loci.

Measures of heterozygosity were not significantly different between the microsatellite dataset and the RAD datasets; average *H*_*O*_ was 0.431 for the microsatellite dataset and 0.392 in RAD_1180, with a range from 0.354 to 0.477 across all RAD datasets (Table 3). Average *H*_*E*_ was 0.388 for the microsatellite dataset and 0.307 for RAD_1180 and ranged from 0.300 to 0.340 for all RAD-Seq datasets (Table 3). Average *F*_*ST*_ for microsatellites was 0.124, which was significantly greater than average *F*_*ST*_ for only one of the RAD datasets—the smallest (RAD_239; Table 3). Within the RAD datasets, average *H*_*O*_ was significantly greater in RAD_25198 than all others, and it was significantly lower in RAD_6255 than in all others; *H*_*O*_ did not predictably increase or decrease as the number of loci increased (Table 3). Additionally, within RAD datasets, average *H*_*E*_ was significantly greater in RAD_25198 than all others. *F*_*ST*_ ranged from 0.046 to 0.108 among the RAD datasets (Table 3). There was no significant difference in *F*_*ST*_ in the three smallest RAD datasets, but the three largest RAD datasets all had increased *F*_*ST*_ relative to the smaller datasets (Table 3). The dataset with the largest value of *F*_*ST*_ was RAD_6255 (Table 3). Average *F*_*IS*_ using microsatellites was not significantly different than *F*_*IS*_ calculated using RAD datasets; within RAD datasets, *F*_*IS*_ generally increased as more loci were added, although RAD_25198 had the lowest value of *F*_*IS*_ (Table 3). Many of the population genetic statistics were disproportionately affected by loci with very low or very high values of *F*_*ST*_, *F*_*IS*_, or heterozygosity (Fig. 2). The effect of extreme loci was particularly evident in the larger datasets (RAD_6255 and RAD_25198), in which there were large numbers of loci with extreme values (e.g., *F*_*ST*_ of 1.0; Fig. 2).

### Pairwise *F*_*ST*_

The values of pairwise *F*_*ST*_ for each sampling location relative to other sampling locations were remarkably consistent across datasets (Table 4). For most sampling locations pairwise *F*_*ST*_ estimated by SSRs was approximately twice as large as RAD dataset estimates. For every dataset, pairwise *F*_*ST*_ between Seahorse Key and all other sampling locations was the highest. For every RAD dataset, Islamorada had the lowest value for pairwise *F*_*ST*_, but the SSR dataset identified West Palm Beach as the sampling location with the lowest estimate of pairwise *F*_*ST*_. Even as the amount of missing data increased, the pairwise *F*_*ST*_ estimates remained consistent; RAD_25198 produced similar values to smaller RAD datasets (Table 4).

**Table 4:**
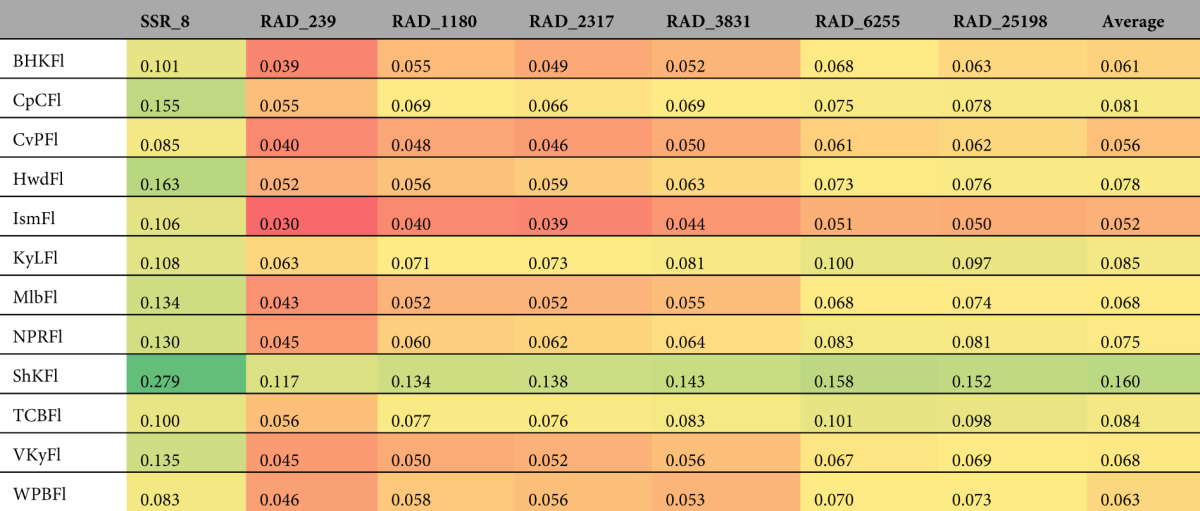
Pairwise *F*_*ST*_ for each sampling location (i.e., one sampling location versus all others) for each of the seven datasets. Within each data set, lower (warmer colors) and higher (cooler colors) values of *F*_*ST*_ are shown using color-coding.

### *F*_*IS*_ by sampling location

Cape Canaveral was the location with the highest *F*_*IS*_ using the microsatellite data (SSR_8), followed by Key Largo and Seahorse Key (Table 5). Meanwhile, for all RAD datasets, Seahorse Key had one of the lowest (i.e., most negative) *F*_*IS*_ values among all populations. Within the RAD datasets, the number of loci and/or amount of missing data affected *F*_*IS*_. For example, in Key Largo, the largest dataset yielded a value of 0.015, while the smallest dataset had a value of −0.194. This was not a large absolute change in *F*_*IS*_, but the interpretation of this statistic changed based on whether it is positive or negative (higher values indicate a greater level of inbreeding). In general, within RAD datasets, *F*_*IS*_ increased as loci were added, although this trend was not universal, especially in the largest RAD dataset. For instance, in Bahia Honda Key, *F*_*IS*_ was lowest in the largest dataset RAD_25198 (25,198 loci, 78.1% missing data). Conversely, in Islamorada, *F*_*IS*_ was lowest in the smallest dataset (RAD_239, 11.7% missing data).

**Table 5:**
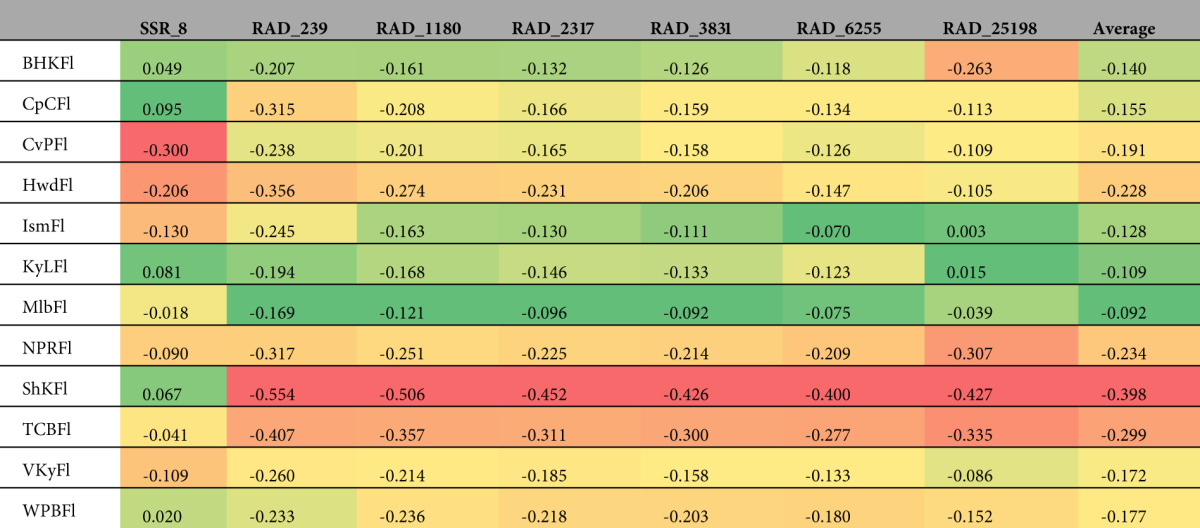
The variation in average inbreeding coefficient (*F*_*IS*_) among data sets and populations. Within each data set, lower (warmer colors) and higher (cooler colors) values of *F*_*IS*_ are shown using color-coding. The average value of *F*_*IS*_ across all data sets for each population is shown in the last column of the table.

### Heterozygosity by sampling location

Observed heterozygosity for each sampling location ranged from 0.320 (Seahorse Key) to 0.451 (Hollywood) when averaged across all datasets (Table 6). For most datasets, Seahorse Key was the sampling location with the lowest *H*_*O*_, although notably RAD_25198 identified six other sampling locations with lower *H*_*O*_ than Seahorse Key (Table 6). Similarly, most datasets reported Hollywood as the sampling location with the highest *H*_*O*_, but SSR_8 found Convoy Point and Islamorada had higher *H*_*O*_ than Hollywood, and RAD_25198 identified five other sampling locations with greater *H*_*O*_, with Key Largo having the highest *H*_*O*_ (Table 6). For most sampling locations, measures of *H*_*O*_, when ranked relative to other sampling locations, remained similar across all RAD datasets except RAD_25198. Interestingly, the values of *H*_*O*_ ranked relative to other sampling locations were more similar between SSR_8 and the five smallest RAD datasets (RAD_239-RAD_6255) than any of the five smallest RAD datasets were to RAD_25198 (Table 6).

**Table 6:**
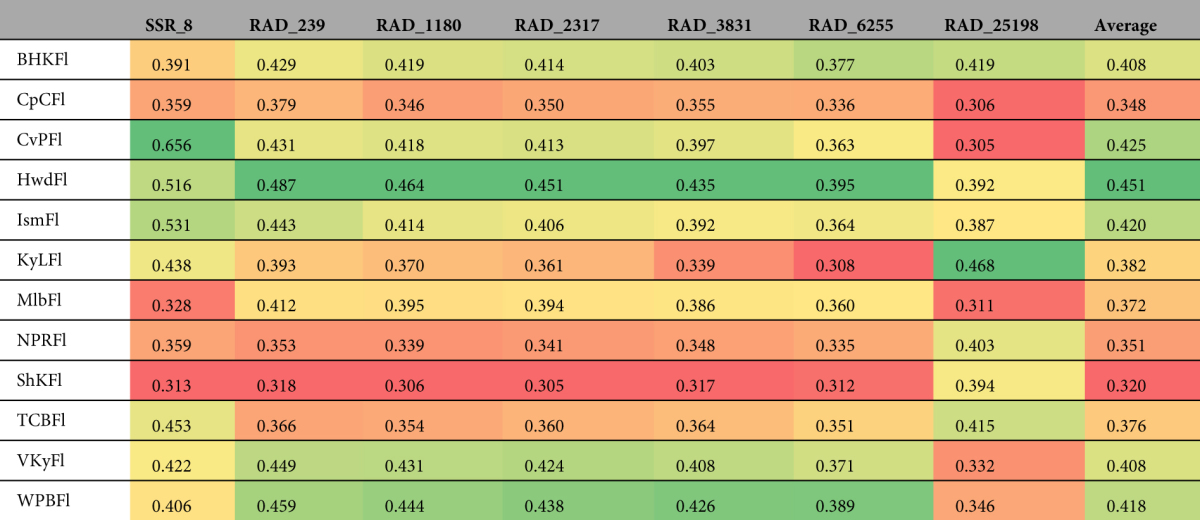
The variation in observed heterozygosity (*H*_*O*_) among data sets and populations. Within each data set, lower (warmer colors) and higher (cooler colors) values of *H*_*O*_ are shown using color-coding. The average value of *H*_*O*_ across all data sets for each population is shown on the bottom row of the table.

### PCA and SVDQuartets

**Figure 3:**
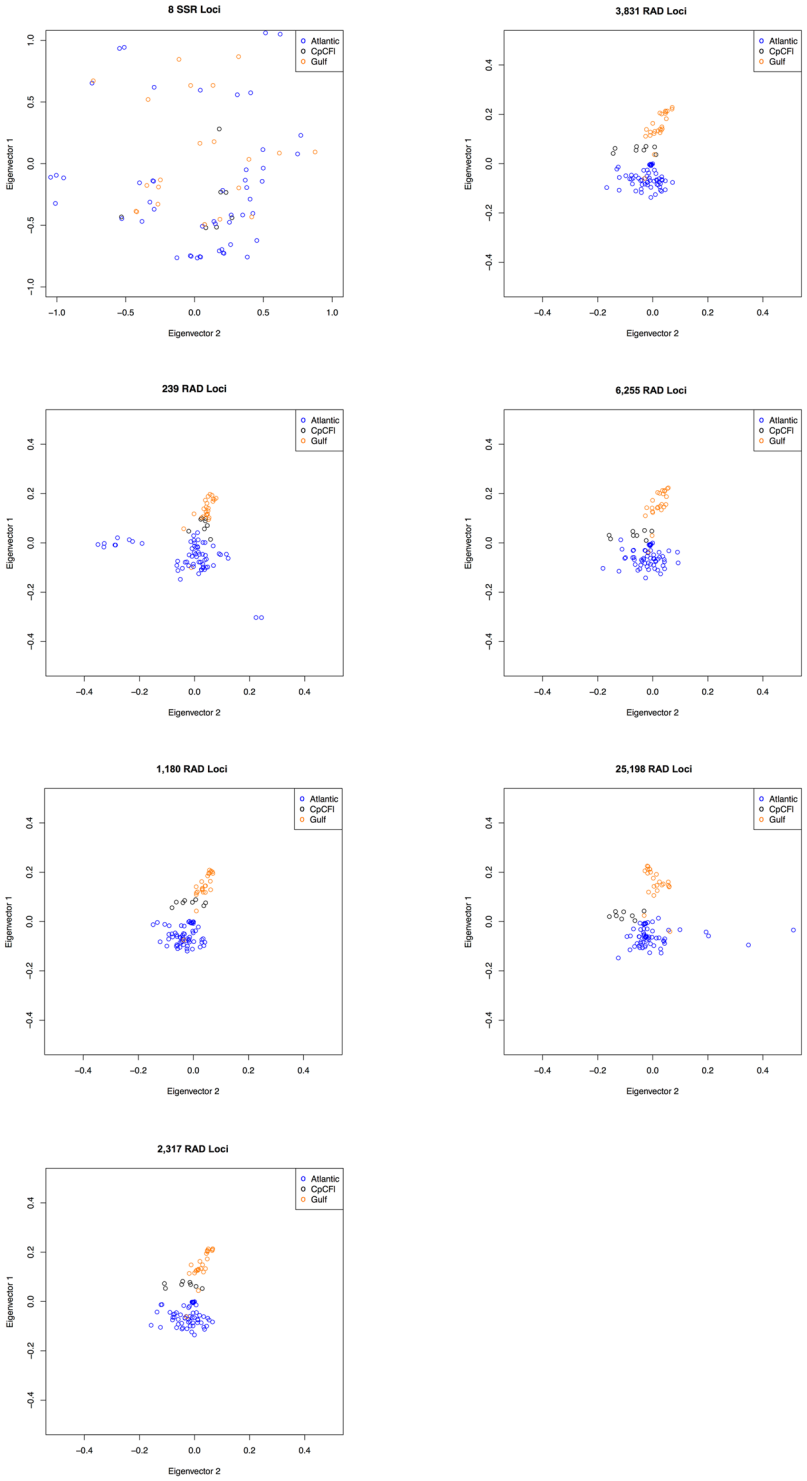
Principle component analysis (PCA) for all seven data sets. Note that the scales of the axes of the SSR_8 plot are different than the axes of all the RAD plots.

The PCA analysis revealed that microsatellite data did not identify clear groupings of individuals based on sampling location or other geographical divisions (Fig. 3). Similarly, RAD_239 did not differentiate the samples into discrete clusters. However, RAD_1180, RAD_2317, RAD_3831, RAD_6255, and RAD_25198 all divided the samples into two groups with minimal overlap in the PCA visualization: one group was Gulf Coast samples, and the other group was Atlantic Coast samples (Fig. 3). Closer inspection of the PCAs revealed that most of the Cape Canaveral individuals formed a discrete cluster intermediate between the two other clusters (Gulf and Atlantic). Most RAD datasets had sufficient resolution to place Cape Canaveral between the Gulf and Atlantic clusters, but the use of a small number of loci (i.e., RAD_239) was unable to show this relationship. Furthermore, the two largest datasets, RAD_6255 and RAD_25198, showed Cape Canaveral individuals clustering more closely to the Atlantic than the Gulf cluster.

**Figure 4:**
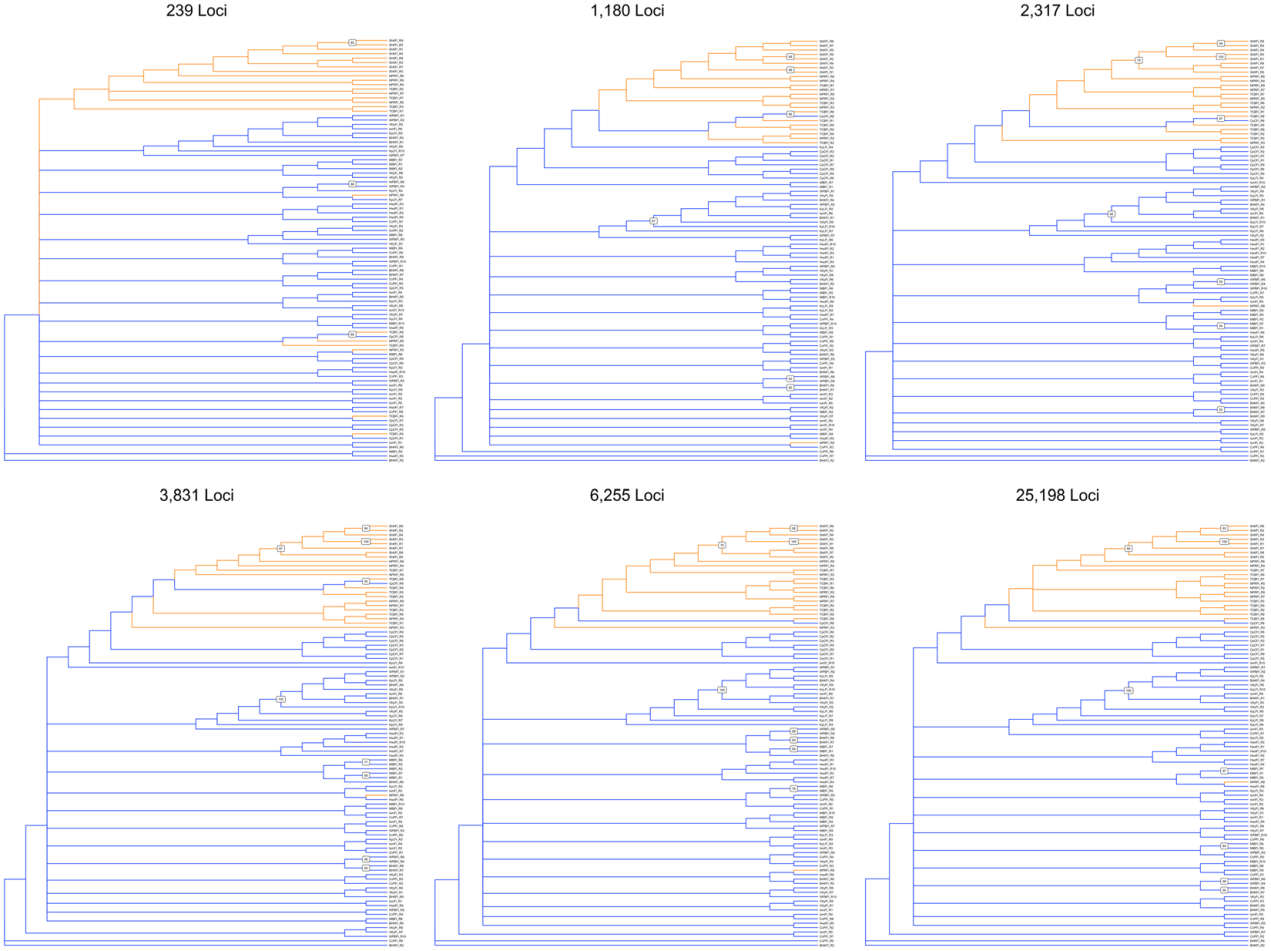
Trees estimated using every individual for each RAD dataset in SVDQuartets. Orange branches indicate individuals from sampling locations in the Gulf of Mexico, and blue branches represent individuals from Atlantic sampling locations.

The 50% majority-rule consensus bootstrap trees generated with SVDQuartets showed substantial variation between datasets when inferring genealogical relationships between individuals and/or sampling locations (Fig. 4). In many cases, dataset RAD_239 did not identify genealogical relationships that were recovered with other datasets with more loci. However, certain key relationships among individuals were consistently shown in multiple datasets with thousands of loci. In every dataset except RAD_239 (i.e., every dataset with at least 1180 loci), all Seahorse Key (ShKFl) samples formed a clade (Fig. 4). In four datasets (RAD_2317, RAD_3831, RAD_6255, RAD_25198), all Gulf Coast (NPRFl, ShKFl, TCBFl) samples (except one individual: NPRFl_R8), together with all Cape Canaveral (CpCFl) samples, formed a clade that is sister to all remaining Atlantic Coast samples plus NPRFl_R8. Interestingly, this relationship was not recovered in RAD_1180, the dataset with ‘ideal’ filtering of loci—but all datasets with more loci (and therefore more missing data) did recover the relationship. Each RAD dataset had nodes with varying levels of bootstrap support (Fig. 4). Datasets with fewer loci showed few nodes with bootstrap support >70%; RAD_239 had three such nodes. More loci resulted in more nodes with bootstrap support >70%, up to a point: RAD_1180 had six highly supported nodes, RAD_2317 had eight, and RAD_3831 had the most with nine. Then the number of highly supported nodes slightly declined as more loci were added: RAD_6255 and RAD_25198 each had eight nodes with bootstrap support >70% (Fig. 4).

### Sampling loci

**Figure 5:**
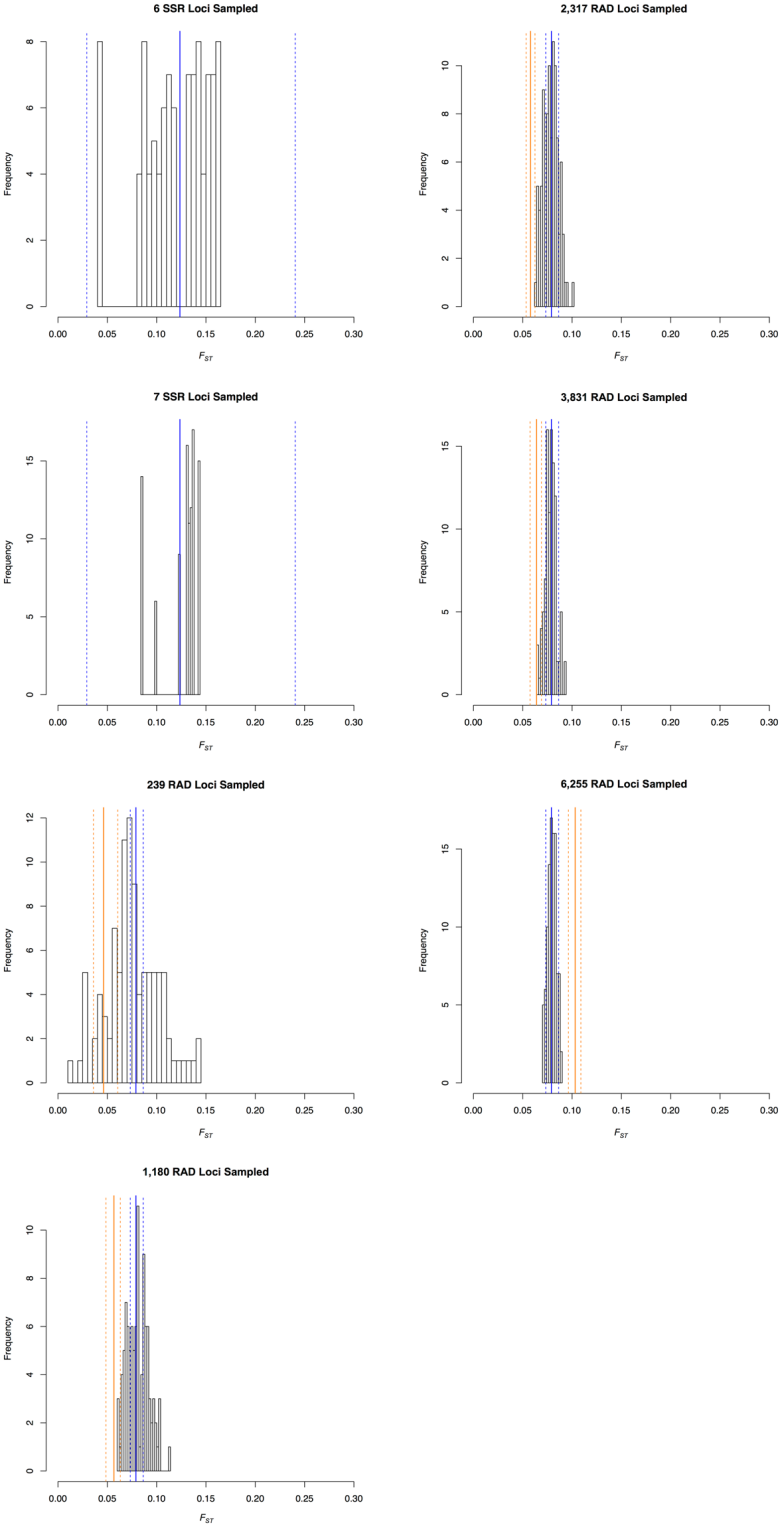
Histograms showing the distribution of the 100 samplings of loci from a larger data set. In the first two panels, six and seven SSR loci, respectively, were randomly sampled 100 times from the SSR_8 data set, and the distribution of the 100 calculations of *F*_*ST*_ are shown. The solid blue line indicates the parameter value estimated using all eight loci, and the dashed blue lines show the 95% confidence interval. In the remaining five plots, the histogram shows parameter estimates using the number of loci (239, 1,180, 2,317, 3,831, and 6,255, respectively) in the data set randomly sampled from RAD_25198 100 times. The solid blue lines indicate the *F*_*ST*_ value estimated using all 25,198 loci, and the dashed blue lines show the 95% confidence interval. The solid orange lines indicate *F*_*ST*_ estimated using the original data set (RAD_239, RAD_1180, RAD_2317, RAD_3831, and RAD_6255, respectively) and dashed orange lines show the 95% confidence interval for this estimate.

Analyzing differently sized samples of loci from RAD_25198 and SSR_8 provided several crucial insights. A microsatellite dataset with seven loci sampled from SSR_8 performed better in estimating *F*_*ST*_ than a dataset with six loci, although in each case, all 100 sampled replicates fell within the 95% confidence interval of *F*_*ST*_ for the complete SSR_8 dataset (Fig. 5). For all RAD datasets, the value of *F*_*ST*_ estimated using only originally filtered data is different from all 100 permuted values of *F*_*ST*_ calculated from an equivalent number of loci sampled from the largest dataset (RAD_25198). For almost all datasets, *F*_*ST*_ based on sampled loci was less than *F*_*ST*_ using original loci, except for one dataset (RAD_6255), *F*_*ST*_ based on the sampled loci was greater. Strikingly, in none of the datasets did the confidence intervals from the sampled loci overlap with the confidence intervals of the estimated *F*_*ST*_ values from the original data (Fig. 5). The percentage of missing data in the largest dataset clearly had an immense impact. Even when very few loci (e.g., 239 loci) were sampled from the largest dataset, the distribution of *F*_*ST*_ values clustered around the estimated *F*_*ST*_ using all 25,198 loci (Fig. 5), indicating that missing data, not number of loci, affected the differences in measured *F*_*ST*_.

## Discussion

### Insights regarding choice of loci

Our results indicate that filtering loci using the standard cutoff (i.e., 75–80% of individuals must possess data for a given locus for that locus to be retained) should not be the gold standard in RAD-Seq studies—it is possible to retain many more loci without inflated statistics^23–28^. *F*_*ST*_ increased as missing data increased, as predicted by simulation studies, but the relationship is more nuanced than previously assumed. *F*_*ST*_ increases as the percentage of missing data increases—up to a point—and then decreases from RAD_6255 to RAD_25198, as the percentage of missing data nearly doubles, from 41.3% to 78.1% (Table 3). When more loci are included, the distribution of *F*_*ST*_ across the genome is more completely sampled. However, adding loci with more missing data may cause analyses to miss low-frequency alleles in the loci with extensive missing data, which would add error to average estimates of *F*_*ST*_. Sampling analyses confirmed that *F*_*ST*_ generally increased as missing data increased (Fig. 5). Heterozygosity was less affected by missing data, as there was little or no change in either observed or expected heterozygosity when the percentage of missing data ranged between 11.7% (RAD_239) and 41.3% (RAD_6255). Only the largest dataset (RAD_25198, with 78.1% missing data) showed significantly higher heterozygosity than other datasets. Some simulation studies reported that missing data could inflate *F*_*ST*_, and would likely inflate estimates of heterozygosity, leading to calls for removing loci with incomplete sampling^13^. However, more recent simulation studies showed that removing loci with higher mutation rates, which are more likely to have missing data, negatively impacted phylogenetic analyses^22^. Our study shows the importance of thoroughly exploring how loci are filtered in empirical systems. Extreme amounts of missing data yield higher estimates of *F*_*ST*_ and heterozygosity and lower estimates of *F*_*IS*_ (Table 3). A large number of loci in RAD_25198 had very high values of certain statistics (e.g., hundreds of loci with *F*_*ST*_ > 0.975 and thousands of loci with *H*_*O*_ > 0.975), which severely impacted average estimates of these statistics (Fig. 2). Notably, not all datasets have these extreme loci—dataset RAD_3831, which only requires 52.1% of individuals to have data for a given locus, and had 29.4% missing data, did not suffer from extreme loci, despite liberal filtering.

Although missing data caused some statistics to increase, it did not dramatically affect our conclusions. For many analyses, using datasets other than RAD_1180, especially RAD_2317 and RAD_3831, did not change the interpretation of the results. Regardless of which of the three datasets was used, *F*_*ST*_ was relatively low—between 0.057 and 0.066. Importantly, nearly doubling the amount of missing data from 17.1% (RAD_1180, the ‘gold standard’) to 29.4% (RAD_3831) resulted in a very small increase in *F*_*ST*_ and no significant change in other statistics (*F*_*IS*_, *H*_*O*_, *H*_*E*_; Table 3). Furthermore, using very few loci (RAD_239) did not significantly change any of the statistics estimated using RAD_1180 (Table 3). Our data indicate that the often-used cutoff of 75–80% individuals with locus data is arbitrary, and different cutoffs should be considered and evaluated on a case-by-case basis to ensure an appropriate number of loci are used. The results suggest that in many cases, only minimal filtering of loci is needed, and many more loci can be retained than typically are. Researchers who wish to maximize the number of loci in their study could likely use very low cutoffs (e.g., require a locus to have data for >10% of individuals).

Typically, microsatellite datasets have lower *F*_*ST*_ values relative to SNPs due to a larger number of alleles, although simulation studies have shown evidence that *F*_*ST*_ can be elevated up to an order of magnitude in microsatellite datasets due to factors such as correlated allele frequencies^38^. Average *F*_*ST*_ ranged from 0.046 to 0.124 across all datasets—so there is not high differentiation detected in any dataset (Table 3). When using any RAD dataset except RAD_239, *F*_*ST*_ calculated using RAD loci was statistically indistinguishable from the microsatellite dataset (Table 3). In theory, a three-to-four-fold change in *F*_*ST*_ could alter biological conclusions—possibly with deleterious results (e.g., identifying populations or management units that would be prioritized for conservation)—but no matter how the loci were filtered, there was a relatively small range of estimated *F*_*ST*_. RAD-Seq studies where larger values of *F*_*ST*_ were detected could exhibit larger absolute changes in *F*_*ST*_ when using different loci filtering cutoffs (e.g., refs^39,40^).

Similarly, the interpretation of *F*_*IS*_ and *H*_*O*_ could impact how data are considered in a biological context (e.g., identifying locations at risk for inbreeding depression). Positive values of *F*_*IS*_ and/or low values of *H*_*O*_ often indicate inbreeding, which means sampling locations are more vulnerable than other sampling locations. As noted earlier, SSR_8 identified five sampling locations with positive *F*_*IS*_ values (Table 5). Only one of these sampling locations (Key Largo) also had positive *F*_*IS*_ values in any of the RAD datasets. Clearly, marker choice (microsatellite versus RAD-Seq) affected the assessment of which populations are more vulnerable based on *F*_*IS*_ values. Agreement between these two markers types would facilitate identifying sampling locations vulnerable to inbreeding depression. However, it is understandable that different markers would lead to different results, as mutation rate can affect estimation of *F*_*IS*_—in theory, as the mutation rate increases, *F*_*IS*_ should decrease. The data showed opposite result though, as *F*_*IS*_ was higher in the SSR_8 dataset for most sampling locations (Table 5). This is likely because estimates of *H*_*O*_ and *H*_*E*_ have larger variance when few loci are used, as in the SSR_8 dataset (Table 3). The relative values of *H*_*O*_ and *H*_*E*_ can dramatically affect the interpretation of F_IS_, especially when *H*_*O*_ and *H*_*E*_ are similar (e.g., using the equation *F*_*IS*_ = (*H*_*E*_ − *H*_*O*_)/*H*_*E*_ would yield *F*_*IS*_ of 0.25 when *H*_*O*_ = 0.4 and *H*_*E*_ = 0.5, but *F*_*IS*_ = −0.25 if *H*_*O*_ and *H*_*E*_ are reversed). Within RAD datasets, estimates of *F*_*IS*_ for each sampling location were fairly consistent and *F*_*IS*_ increased as missing data increased, but this trend was not universal (Table 3). Identifying vulnerable sampling locations based on *H*_*O*_ revealed that RAD_25198 led to different conclusions than most other datasets. Across datasets, measures of *H*_*O*_ within each sampling location were consistent relative to other sampling locations, except for RAD_25198 (Table 6). Missing data impacted this analysis; a large number of loci in RAD_25198 had either very high or very low *H*_*O*_, possibly leading to the pattern of *H*_*O*_ in RAD_25198 that contrasted with patterns in virtually every other dataset (Table 6, Fig. 2).

The PCA results show that as the number of loci increases, the definition of clusters improves, plateauing with RAD_2317 or RAD_3831. The clustering is similar in all RAD datasets with 1,180 or more loci, with Cape Canaveral individuals falling between the Gulf and Atlantic clusters. As more loci are added, the Cape Canaveral samples appear to be closer to the Atlantic cluster, especially in datasets RAD_6255 and RAD_25198 (Fig. 3). Taking into account the SVDQuartets results clarifies the clustering—all Cape Canaveral samples form a clade with all Gulf samples except one. However, this relationship is only present in datasets with 2,317 loci or greater—the putatively ‘gold standard’ dataset RAD_1180 does not show this relationship.

### Phylogeographic patterns in red mangroves

Based on previous studies using microsatellite data^32,36^, the relationship of Cape Canaveral samples to other sampling locations, as found here with both PCA and SVDQuartet analyses of RAD-Seq data, was surprising—previous studies did not find that any of the individuals in the Cape Canaveral population clustered with any of the Gulf samples. These new data could indicate an Atlantic-Gulf phylogeographic discontinuity, and that Cape Canaveral is an anomaly due to a lack of phylogeographic resolution, recent population founding, or human-mediated transplantation. The intermediate placement of Cape Canaveral in many of the PCAs suggests that it may actually cluster with the Atlantic samples, especially when considering datasets RAD_6255 and RAD_25198, indicating a phylogeographic break (Fig. 3). However, the SVDQuartets results place Cape Canaveral in a clade with the vast majority of Gulf samples, although this relationship is not highly supported in any datasets (i.e., bootstrap support is not >70% for this clade in any dataset) (Fig. 4). Assuming that Cape Canaveral is more closely related to Gulf samples, the age of the divergence between the two clades (Atlantic, Gulf + CpCFl) comes into question. Northern Florida represents the northern limit of the range of red mangroves^33^. Typically, populations of these trees in northern Florida are periodically extirpated due to freezing events, and these areas are re-colonized. The lower values of *H*_*O*_ in northern populations (CpCFl, MlbFl, ShKFl, NPRFl, TCBFl) relative to southern populations indicate that these populations were likely founded more recently from a small number of propagules. The Cape Canaveral population was likely founded by individuals from the Gulf Coast, suggesting that the divergence between the two clades (Atlantic, Gulf + CpCFl) is very recent.

Previous research indicates that gene flow is greater from the Gulf Coast to the Atlantic Coast in red mangroves; there may be ongoing gene flow from the Gulf to Cape Canaveral^32^. Alternatively, alleles from the Gulf Coast could have migrated into an existing Cape Canaveral population and proliferated due to other processes (e.g., drift). Another explanation for the sister relationship between the Gulf samples and Cape Canaveral is human-mediated transplantation of propagules or seedlings from the Gulf Coast to Cape Canaveral. However, all available publications and information from land managers who replied to requests for information confirm that any restoration that required importation of propagules used either local propagules or seedlings from the southern Atlantic Coast (ref.^41^, personal communication with Rangers from Cape Canaveral National Seashore). Another possible explanation for this result is that red mangrove propagules were accidentally transported from the Gulf Coast of Florida to Cape Canaveral during construction of the Kennedy Space Center in the 1960s. Construction of the Space Center was a massive project. It is noteworthy that nearly 100,000 tons of steel was transported from the Gulf Coast to Cape Canaveral in numerous trucks; the transport of a few mangrove propagules during this process could easily have established a Gulf genotype in the Cape Canaveral area^42^. We conclude that, in contrast to microsatellites, RAD datasets recover a relationship between the Gulf Coast and the Atlantic Coast (excluding Cape Canaveral) that supports the presence of a maritime discontinuity in red mangroves. However, as red mangroves can disperse long distances, a population or populations that recently established in Cape Canaveral likely had a founder or founders that were predominantly of Gulf Coast origin. The fact that previous studies using SSRs did not elucidate this relationship is not surprising—both the PCA analysis and SVDQuartets analysis indicate that 1180 loci were barely sufficient to infer the placement of Cape Canaveral—datasets with many more loci were needed (Figs 3 and 4). The large number of loci required to resolve such relationships highlights why liberal filtering of RAD-Seq loci is advisable.

## Conclusions

We cannot overemphasize the importance of thoroughly exploring RAD-Seq datasets when performing phylogeographic analyses—it is too easy to jump to conclusions when only using one arbitrary cutoff to filter loci. Our empirical data confirm that estimates of *F*_*ST*_ and/or heterozygosity may become inflated as missing data increase. However, this does not happen as quickly as implied in simulation studies as loci with missing data are added—liberal filtering of loci retains loci valuable for phylogeographic or phylogenetic inference, without inflating population genetic statistics. Thus, regardless of the cutoff value used to filter loci, researchers should investigate several other cutoffs with both increased and decreased amounts of missing data to appreciate fully the impact of missing data on parameters in their study. We found no evidence that the 75% or 80% cutoff commonly employed was optimal. In many analyses, other datasets with cutoffs ranging from 31.3% to 67.7% performed just as well as or better than RAD_1180. Many RAD-Seq studies aim to multiplex as many individuals as possible in a HTS run; our results show that retaining loci with more missing data is feasible and advantageous in empirical studies, and that researchers can include more samples in a single sequencing run. Our study confirmed that microsatellites were a valuable tool for inexpensively estimating population genetic statistics, such as *F*_*ST*_, *F*_*IS*_, and heterozygosity. However, this study revealed that the thousands of additional loci from across the genome provided by RAD-Seq increased phylogeographic resolution. We found that red mangroves likely have a phylogeographic discontinuity at the southern tip of Florida that was not detected in previous studies using SSRs^32,36^ and that a single population from the Atlantic coast of Florida arose via recent colonization by propagules (either natural or human-mediated) from the Gulf coast.

## Methods and Materials

### Sample collection, DNA isolation

We collected leaf tissue from plants of *R. mangle* from 12 locations in Florida (Fig. 1). At each location, we collected one leaf from 10–20 individuals that were spaced at least 15 m apart to minimize collecting closely related individuals. For each sampling location, we randomly selected 8 individuals to use in genetic analyses. GPS coordinates for each sampling location were recorded (Table 1). Each sampled leaf was placed in a labeled bag with silica gel and stored for 1–12 months; we then extracted DNA from the dried leaf tissue using a standard CTAB protocol^43^.

### Microsatellite amplification and analysis

We PCR-amplified eight nuclear microsatellite loci for *R. mangle* (RM 11, 19, 21, 36, 38, 41, 46, 47)^37^. An M13 protocol^44^ was used to label amplicons with four fluorescent dyes (6-FAM, NED, PET, VIC). The PCR (25-μL reactions) contained: 5X buffer (5 μL), 2.5 mM MgCl_2_ (2 μL), 2.5 mM dNTP (0.5 μL), 0.12 μM forward primer with M13 label attached (1.25 μL), 4.5 μM reverse primer (1.25 μL), 4.5 μM fluorescent dye (2.5 μL), H_2_O (10 μL), Taq polymerase (0.5 μL), and 50 ng template DNA (2 μL). We carried out PCR in a Biometra T3 Thermocycler (Whatman Biometra, Goettingen, Germany) using the following cycles: initial denaturing at 94 °C for 3 minutes; 35 cycles of 94 °C (45 seconds), 52 °C (45 seconds), 72 °C (75 seconds); final elongation at 72 °C for 20 minutes. We used the Applied Biosystems 3730 DNA Analyzer (Applied Biosystems, Foster City, United States) at the University of Florida Interdisciplinary Center for Biotechnology Research to detect the fluorescent peaks. We determined microsatellite peaks in Geneious 6.5 (http://www.geneious.com/) using the GeneScan 600 size standard ladder for calibration^45^.

### RAD-Seq library preparation and data processing

We followed the double-digest RAD-Seq protocol developed by Peterson, *et al*.^46^. For each sample, we constructed 96 DNA libraries by digesting approximately 200 ng genomic DNA with *Eco*RI and *Mse*I. We then ligated Illumina adapters and unique 8–10-nucleotide barcodes to the DNA fragments. The DNA libraries were PCR-amplified in two separate reactions and pooled to minimize early PCR bias. We size selected 250–450-bp fragments using gel electrophoresis and sequenced the DNA fragments using the 1 × 100-bp setting on the Illumina HiSeq. 2500 platform. Raw sequence data were deposited in the NCBI Sequence Read Archive (accession numbers pending). We processed the raw Illumina reads using the FAST-X toolkit (http://hannonlab.cshl.edu/fastx_toolkit/) to filter sequences; we required 95% of bases to be above a quality score of 30 to retain a read. We then converted the sequences from FASTQ to FASTA, demultiplexed the reads, sorted them by barcodes, and trimmed the sequences by removing the final 2 bases to ensure that we were using only high-quality sequence data. We assembled the sequences into loci using the STACKS 1.24 pipeline^47^ with the following parameter settings: -n 3 -m 3 -M 2 (parameters were optimized following Mastretta-Yanes, *et al*.^29^); all other parameters were left as the default. We selected seven datasets (one microsatellite and six RAD-Seq) and used a variety of analyses to compare the results produced by each dataset (Table 2). We used the ‘populations’ program in STACKS to produce an unfiltered dataset of RAD-Seq loci using the ‘write single SNP’ command and requiring a minor allele frequency >0.05. We then removed human, fungal, and microbial contamination from the loci and filtered loci by representation across individuals using an R script to create five smaller datasets (Data_aquisition.R; this script and all other scripts are available at https://github.com/richiehodel/red_mangrove_RAD_SSR). Filtered datasets were required to have locus data for a certain number of individuals for the given locus to be retained in the analysis; the number of individuals could range from 1–96 (Supplemental Fig. 1). The datasets were chosen such that they encompassed a wide range of loci and missing data.

### Population genetic analyses

We used an R script (Basic_stats.R) and the R package ‘hierfstat’^48^ to calculate average *F*_*ST*_, the inbreeding coefficient *F*_*IS*_, *H*_*O*_, and *H*_*E*_ for each of the seven datasets. To investigate how the number of loci affected comparisons of population genetic statistics among populations, we calculated pairwise *F*_*ST*_ (one sampling location versus all others combined) for each sampling location for each dataset using GenoDive^49^ and an R script (Pairwise_Fst.R). Additionally, we calculated *F*_*IS*_ and *H*_*O*_ for each sampling location for each dataset to determine how measures that often inform conservation practices might be affected by the number of loci and amount of missing data. We measured how missing data were partitioned across sampling locations to verify that there were not any sampling locations with unusually high or low amounts of missing data (Table 1). Additionally, we investigated how several population genetic statistics were distributed across loci in each of the datasets (Stat_Distribution.R; Fig. 2).

### Principal components and SVDQuartets

We used a principal component analysis (PCA) implemented in the R package ‘SNPRelate’^50^ to identify clusters of individuals in the RAD data with an R script (VCF_PCA.R) and GenoDive to run a PCA on the microsatellite data. After visualizing the initial results, we tested several ways of grouping sampling locations together based on geography. We used SVDQuartets^51^ to determine genealogical relationships among individuals. This program selects the optimal topology for a quartet of taxa, and, after sampling millions of quartets, infers a phylogeny for all individuals based on choosing the quartets with the best scores and assembling them into a phylogenetic tree. We used an R script (Nexus_creation.R) to convert the output from the ‘populations’ program in STACKS into nexus files that could be read for the SVDQuartets analysis. For each RAD dataset, we evaluated all possible quartets and selected trees under the multispecies coalescent using QFM (Quartet Fiduccia Mattheyses) quartet assembly^52^. We used non-parametric bootstrapping (100 replicates for each dataset) to assess confidence in inferred genealogical relationships between individuals. The R script Tree_formatting.R was used to visualize and annotate the 50% majority-rule trees from SVDQuartets using the R packages ‘ape’^53^ and ‘ggtree’^54^.

### Sampling loci

To test whether the number of loci or percentage of missing data for the loci used is the more important factor impacting measures of fixation, population differentiation, and heterozygosity, we randomly sampled from RAD_25198 (the RAD-Seq dataset comprising 25,198 loci) the equivalent number of loci contained in RAD_239, RAD_1180, RAD_2317, RAD_3831, and RAD_6255, respectively, and used these five sets of sampled loci in analyses. We used an R script (Subsample.R) to randomly sample loci without replacement from RAD_25198 and repeated the sampling 100 times for each dataset. We compared measures of *F*_*ST*_ calculated using the original datasets with results calculated using the sampled loci from RAD_25198 (Fig. 5). We used bootstrapping to calculate 95% confidence intervals around *F*_*ST*_ for the original datasets and for the sets of loci sampled from RAD_25198 (Fig. 5).

### Data availability

The datasets generated during the current study are available in the NCBI Genbank repository, https://www.ncbi.nlm.nih.gov/bioproject/PRJNA397667 (accession numbers SRR5918296-SRR5918355).

## Electronic supplementary material

Supplementary Figure 1

## References

[1] Guichoux E (2011). Current trends in microsatellite genotyping. Mol. Ecol. Resour..

[2] Kalia RK, Rai MK, Kalia S, Singh R, Dhawan AK (2010). Microsatellite markers: an overview of the recent progress in plants. Euphytica.

[3] Hodel, R. G. J. *et al*. The report of my death was an exaggeration: A review for researchers using microsatellites in the 21st century. *Appl. Plant Sci*. **4**, (2016). 10.3732/apps.1600025 PMC4915923 27347456

[4] Seeb JE (2011). Single-nucleotide polymorphism (SNP) discovery and applications of SNP genotyping in nonmodel organisms. Mol. Ecol. Resour..

[5] Gardner MG, Fitch AJ, Bertozzi T, Lowe AJ (2011). Rise of the machines–recommendations for ecologists when using next generation sequencing for microsatellite development. Mol. Ecol. Resour..

[6] Hodel, R. G. J. *et al*. A new resource for the development of SSR markers: Millions of loci from a thousand plant transcriptomes. *Appl. Plant Sci*. **4**, (2016). 10.3732/apps.1600024 PMC4915922 27347455

[7] Andrews KR, Good JM, Miller MR, Luikart G, Hohenlohe PA (2016). Harnessing the power of RADseq for ecological and evolutionary genomics. Nat. Rev. Genet..

[8] Baird NA (2008). Rapid SNP discovery and genetic mapping using sequenced RAD markers. PLoS One.

[9] Smith AM (2010). Highly-multiplexed barcode sequencing: an efficient method for parallel analysis of pooled samples. Nucleic Acids Res..

[10] Andolfatto P (2011). Multiplexed shotgun genotyping for rapid and efficient genetic mapping. Genome Res..

[11] Sonah H (2013). An improved genotyping by sequencing (GBS) approach offering increased versatility and efficiency of SNP discovery and genotyping. PLoS One.

[12] Rafalski A (2002). Applications of single nucleotide polymorphisms in crop genetics. Curr. Opin. Plant Biol..

[13] Arnold B, Corbett-Detig RB, Hartl D, Bomblies K (2013). RADseq underestimates diversity and introduces genealogical biases due to nonrandom haplotype sampling. Mol. Ecol..

[14] Etter, P. D., Bassham, S., Hohenlohe, P. A., Johnson, E. A. & Cresko, W. A. SNP discovery and genotyping for evolutionary genetics using RAD sequencing in *Methods in Molecular Biology* 157–178 (Clifton, 2012). 10.1007/978-1-61779-228-1_9 PMC3658458 22065437

[15] Xu P (2014). Population genomic analyses from low-coverage RAD-Seq data: a case study on the non-model cucurbit bottle gourd. Plant J..

[16] Lowry DB (2017). Breaking RAD: an evaluation of the utility of restriction site-associated DNA sequencing for genome scans of adaptation. Mol. Ecol. Resour..

[17] Davey JW (2013). Special features of RAD sequencing data: implications for genotyping. Mol. Ecol..

[18] Gautier M (2013). The effect of RAD allele dropout on the estimation of genetic variation within and between populations. Mol. Ecol..

[19] Liu N, Chen L, Wang S, Oh C, Zhao H (2005). Comparison of single-nucleotide polymorphisms and microsatellites in inference of population structure. BMC Genet..

[20] Coates BS (2009). Comparative performance of single nucleotide polymorphism and microsatellite markers for population genetic analysis. J. Hered..

[21] Schopen GCB, Bovenhuis H, Visker MHPW, van Arendonk JAM (2008). Comparison of information content for microsatellites and SNPs in poultry and cattle. Anim. Genet..

[22] Huang H, Knowles LL (2016). Unforeseen consequences of excluding missing data from Next-Generation Sequences: Simulation study of RAD sequences. Syst. Biol..

[23] Catchen J (2013). The population structure and recent colonization history of Oregon threespine stickleback determined using restriction-site associated DNA-sequencing. Mol. Ecol..

[24] Jackson AM (2014). Population structure and phylogeography in Nassau grouper (*Epinephelus striatus*), a mass-aggregating marine fish. PLoS One.

[25] Bernardi G, Azzurro E, Golani D, Miller MR (2016). Genomic signatures of rapid adaptive evolution in the bluespotted cornetfish, a Mediterranean Lessepsian invader. Mol. Ecol..

[26] Blanco-Bercial L, Bucklin A (2016). New view of population genetics of zooplankton: RAD-seq analysis reveals population structure of the North Atlantic planktonic copepod *Centropages typicus*. Mol. Ecol..

[27] Rodríguez-Ezpeleta N (2016). Population structure of Atlantic mackerel inferred from RAD-seq-derived SNP markers: effects of sequence clustering parameters and hierarchical SNP selection. Mol. Ecol. Resour..

[28] Van Wyngaarden M (2017). Identifying patterns of dispersal, connectivity and selection in the sea scallop, *Placopecten magellanicus*, using RADseq-derived SNPs. Evol. Appl..

[29] Mastretta-Yanes A (2015). Restriction site-associated DNA sequencing, genotyping error estimation and *de novo* assembly optimization for population genetic inference. Mol. Ecol. Resour..

[30] Bradbury IR (2015). Transatlantic secondary contact in Atlantic Salmon, comparing microsatellites, a single nucleotide polymorphism array and restriction-site associated DNA sequencing for the resolution of complex spatial structure. Mol. Ecol..

[31] Jeffries DL (2016). Comparing RADseq and microsatellites to infer complex phylogeographic patterns, an empirical perspective in the Crucian carp, *Carassius carassius*, L. Mol. Ecol..

[32] Hodel RGJ, Cortez MB, de S, Soltis PS, Soltis DE (2016). Comparative phylogeography of black mangroves (*Avicennia germinans*) and red mangroves (*Rhizophora mangle*) in Florida: Testing the maritime discontinuity in coastal plants. Am. J. Bot..

[33] Tomlinson, P. B. *The Botany of Mangroves* (Cambridge University Press, 2016).

[34] Avise, J. C. Phylogeography: The History and Formation of Species (Harvard University Press, 2000).

[35] Soltis D, Morris A, McLachlan JS, Manos PS, Soltis PS (2006). Comparative phylogeography of unglaciated eastern North America. Mol. Ecol..

[36] Kennedy JP (2017). Contrasting genetic effects of red mangrove (*Rhizophora mangle* L.) range expansion along West and East Florida. J. Biogeogr..

[37] Fu R, Dey DK, Holsinger KE (2005). Bayesian models for the analysis of genetic structure when populations are correlated. Bioinformatics.

[38] Rosero-Galindo C, Gaitan-Solis E, Cárdenas-Henao H, Tohme J, Toro-Perea N (2002). Polymorphic microsatellites in a mangrove species, *Rhizophora mangle* L.(Rhizophoraceae). Mol. Ecol. Notes.

[39] Kang J, Ma X, He S (2017). Population genetics analysis of the Nujiang catfish *Creteuchiloglanis macropterus* through a genome-wide single nucleotide polymorphisms resource generated by RAD-Seq. Sci. Rep..

[40] Manthey JD, Geiger M, Moyle RG (2017). Relationships of morphological groups in the northern flicker superspecies complex (*Colaptes auratus* & *C. chrysoides*). Syst. Biodivers..

[41] Johnson, L.K. & Herren, L.W. Re-establishment of fringing mangrove habitat in the Indian River Lagoon 19–22 (Florida Department of Environmental Protection, 2008).

[42] NASA Public Affairs. *The Kennedy Space Center Story* (Graphic House, 1991).

[43] Doyle JJ, Doyle JL (1987). A rapid DNA isolation procedure for small quantities of fresh leaf tissue. Phytochem. Bull..

[44] Schuelke M (2000). An economic method for the fluorescent labeling of PCR fragments. Nat. Biotechnol..

[45] Kearse M (2012). Geneious Basic: an integrated and extendable desktop software platform for the organization and analysis of sequence data. Bioinformatics.

[46] Peterson, B. K., Weber, J. N., Kay, E. H., Fisher, H. S. & Hoekstra, H. E. Double Digest RADseq: An inexpensive method for de novo SNP discovery and genotyping in model and non-model species. *PLoS One* **7**, (2012). 10.1371/journal.pone.0037135 PMC3365034 22675423

[47] Catchen J, Hohenlohe PA, Bassham S, Amores A, Cresko WA (2013). Stacks: an analysis tool set for population genomics. Mol. Ecol..

[48] Goudet J (2005). hierfstat, a package for R to compute and test hierarchical F-statistics. Mol. Ecol. Notes.

[49] Meirmans PG, Van Tienderen PH (2004). Genotype and Genodive: two programs for the analysis of genetic diversity of asexual organisms. Mol. Ecol. Notes.

[50] Zheng X (2012). A high-performance computing toolset for relatedness and principal component analysis of SNP data. Bioinformatics.

[51] Chifman J, Kubatko L (2014). Quartet inference from SNP data under the coalescent model. Bioinformatics.

[52] Reaz R (2014). Accurate phylogenetic tree reconstruction from quartets: A heuristic approach. PLoS One.

[53] Paradis E, Claude J, Strimmer K (2004). APE: analyses of phylogenetics and evolution in R language. Bioinformatics.

[54] Yu G, Smith DK, Zhu H, Guan Y, Lam TTY (2017). ggtree: an R package for visualization and annotation of phylogenetic trees with their covariates and other associated data. Methods in Ecology and Evolution.

