# Supplementary material for: Adding loci improves phylogeographic resolution in red mangroves despite increased missing data: comparing microsatellites and RAD-Seq and investigating loci filtering
Source: Sci Rep. 2017 Dec 14;7:17598. doi: 10.1038/s41598-017-16810-7 (PMC5730610; doi:10.1038/s41598-017-16810-7)

Adding loci improves phylogeographic resolution in red mangroves despite increased missing data: comparing microsatellites and RAD-Seq and investigating loci filtering

^*^Richard G.J. Hodel^1,2^

Shichao Chen^2,3^

Adam C. Payton^1^

Stuart F. McDaniel^1^

Pamela S. Soltis^2,4^

Douglas E. Soltis^1,2,4^

^1^ Department of Biology

University of Florida

Gainesville, FL 32611, USA

^2^ Florida Museum of Natural History

University of Florida

Gainesville, FL 32611, USA

^3^ College of Life Sciences and Technology

Tongji University

Shanghai 200092, China

^4^ The Genetics Institute

University of Florida

Gainesville, FL 32610, USA

^*^ Corresponding author; email: richiehodel@gmail.com

**Supplementary Figure 1.** First panel: the estimated value of *F_ST_* (hollow black circles) using every possible cutoff point (1-96 individuals); vertical red lines indicate the number of individuals that were used as the cutoff point for the six datasets investigated in depth. Second panel: the estimated value of *F_ST_* (hollow black circles) for every possible number of loci that result from using all cutoffs for filtering, with vertical red lines showing the number of loci associated with the six datasets that were thoroughly investigated. Panels three and four show similar information for *F_IS_*, and panels five and six show estimated values of *H_O_*.


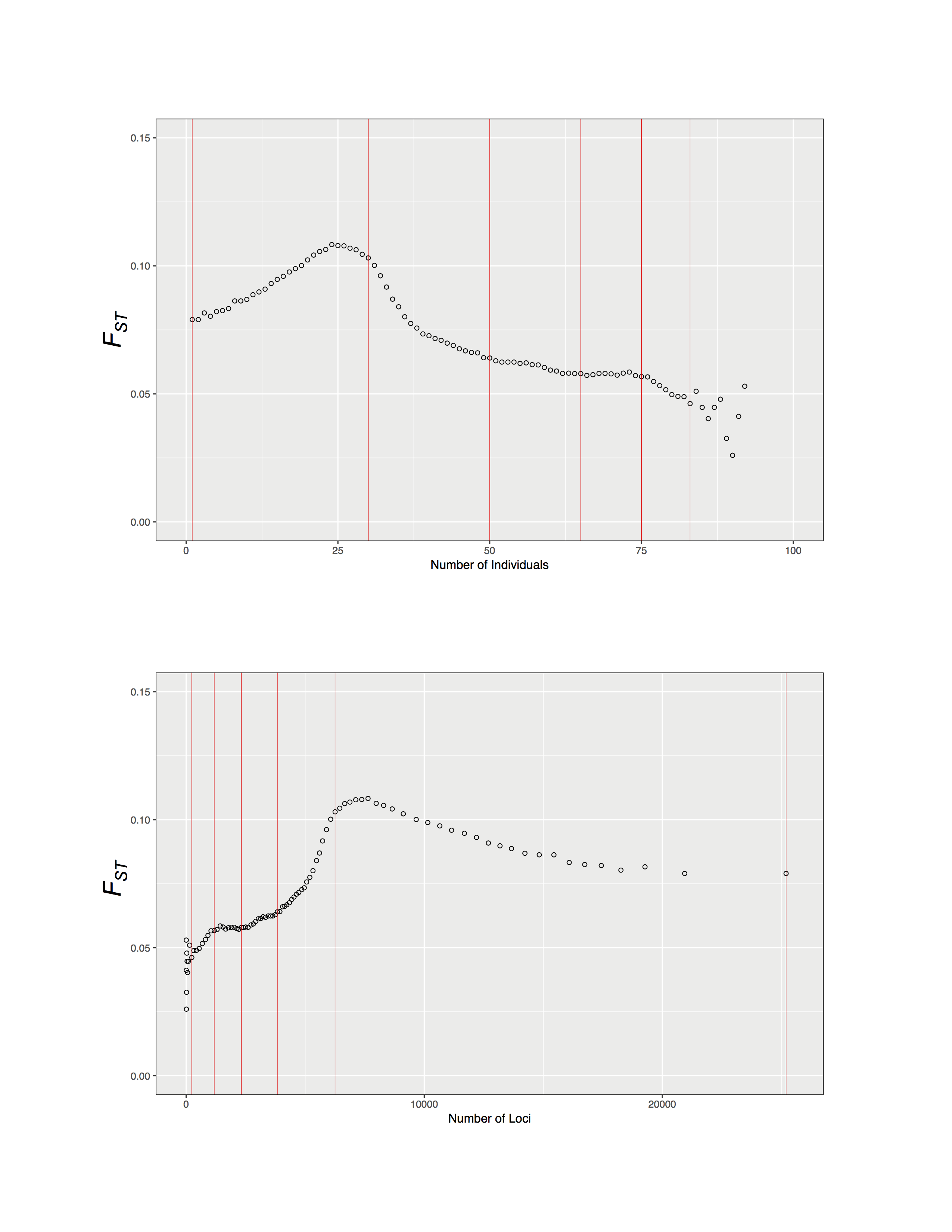


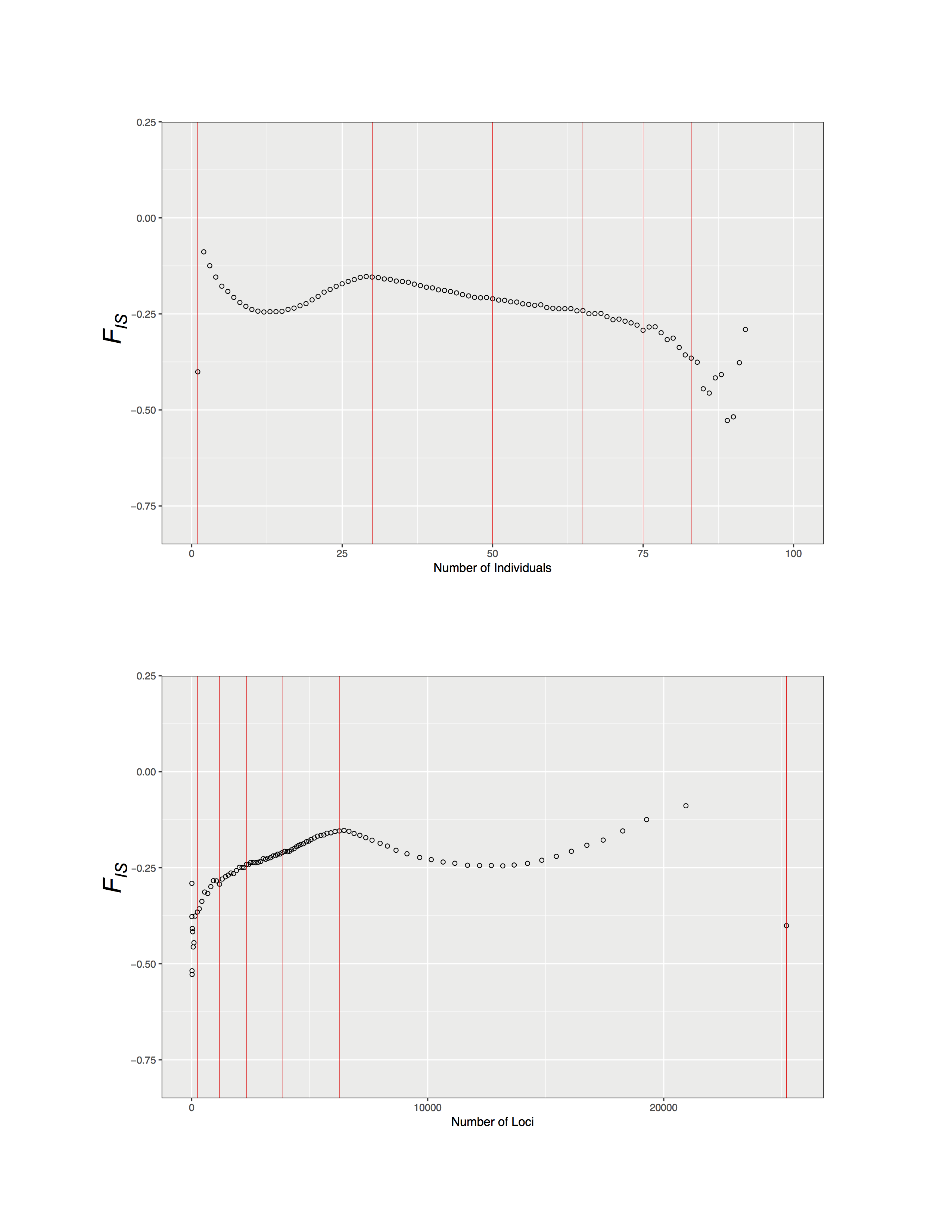


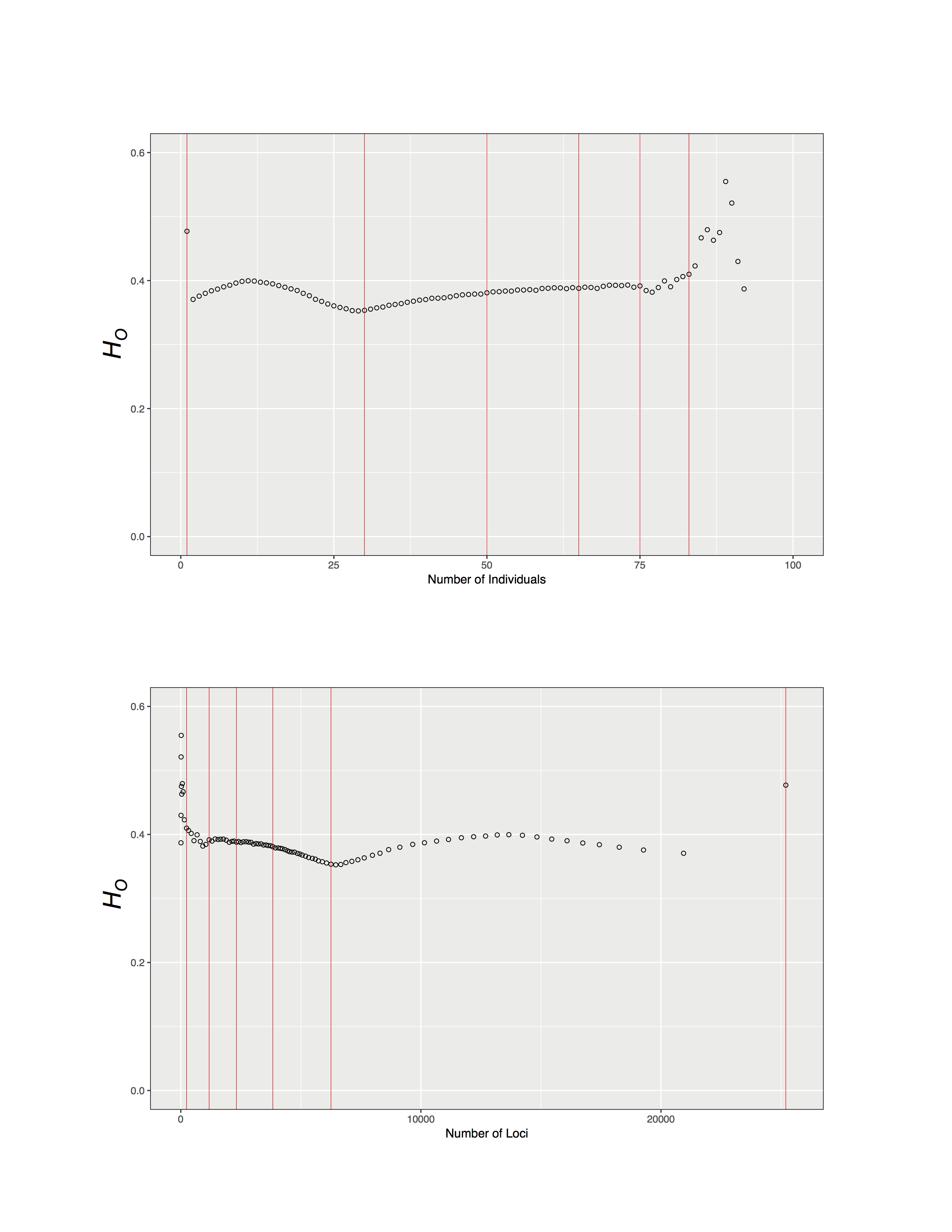

Supplement: Supplementary file 1 — Supplementary Figure 1 [file 41598_2017_16810_MOESM1_ESM.doc]
